# *BRAFV600E* mutation is associated with aggressive features in papillary thyroid carcinomas ≤ 1.5 cm

**DOI:** 10.1186/s40463-021-00543-9

**Published:** 2021-11-06

**Authors:** Jennifer A. Silver, Mariya Bogatchenko, Marc Pusztaszeri, Véronique-Isabelle Forest, Michael P. Hier, Ji Wei Yang, Michael Tamilia, Richard J. Payne

**Affiliations:** 1 Faculty of Medicine, McGill University, Montreal, QC Canada; 2 Department of Otolaryngology-Head and Neck Surgery, McGill University, 3755 Chemin de la Côte-Sainte-Catherine, Montréal, QC H3T 1E2 Canada; 3 Department of Pathology, McGill University, Montreal, QC Canada; 4 Division of Endocrinology, McGill University, Montreal, QC Canada; 5 Sir Mortimer B. Davis-Jewish General Hospital, McGill University, Montreal, QC Canada

**Keywords:** Papillary thyroid microcarcinoma, *BRAF V600E* mutation, Clinicopathologic features, Thyroid cancer

## Abstract

**Background:**

While some studies suggest that the *BRAF V600E* mutation correlates with a high-risk phenotype in papillary thyroid microcarcinoma (PTMC), more evidence is necessary before this mutation can be used to help guide decision making in the management of small thyroid nodules. This study investigated whether *BRAF V600E* mutation is associated with aggressive features in PTMC (≤ 1 cm) and small PTC (1–1.5 cm).

**Methods:**

Retrospective chart review was performed on 121 patient cases. Patients who underwent thyroid surgery for PTMC (≤ 1 cm) or small PTC (1–1.5 cm) were included if molecular testing was done for *BRAF V600E* mutation. Two study groups were created based on tumour size: PTMC (n = 55) and small PTC (n = 66). The groups were analysed for the presence of a *BRAF V600E* mutation and aggressive features, including macroscopic extrathyroidal extension (ETE), lymph node metastasis (LNM), and high-risk histological features (tall cell, columnar cell, hobnail, solid/trabecular, and diffuse sclerosing). The Fischer exact test was used to calculate statistical significance.

**Results:**

*BRAF V600E* mutations were detected in 43.6% of PTMC and 42.4% of small PTC. Of the mutated PTMC nodules, 54.1% demonstrated aggressive characteristics as compared to 19.4% of the non-mutated PTMCs (*p* = 0.010). Of the mutated small PTC tumours, 82.1% had aggressive features. In contrast, 28.9% of the non-mutated small PTCs showed aggressive features (*p* < 0.001).

**Conclusions:**

Our findings demonstrate an association between a *BRAF V600E* mutation and aggressive features in PTMC (≤ 1 cm) and small PTC (1–1.5 cm). Therefore, determining the molecular status of these thyroid nodules for the presence of *BRAF V600E* can help guide patient management.

**Graphical Abstract:**

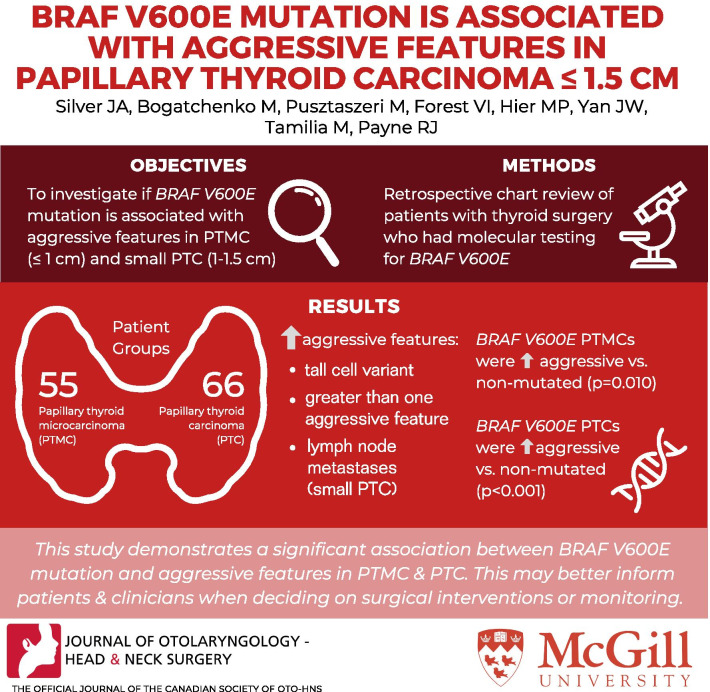

**Supplementary Information:**

The online version contains supplementary material available at 10.1186/s40463-021-00543-9.

## Introduction

Thyroid cancer has become increasingly prevalent in North America, with an incidence in the United States of 13.7 cases per 100,000 in 2017 compared to only 4.9 cases per 100,000 in 1977 [1]. In 2019, thyroid cancer was the fifth most common malignancy diagnosed in women in Canada. It was the leading cancer diagnosed among the ages 15–29 [2]. The most common form of thyroid cancer is papillary thyroid carcinoma (PTC), making up approximately 80% of diagnoses [3, 4].The surge in documented cases can be attributed to recent advances in imaging modalities that can more accurately identify small nodules in the thyroid gland. As such, many thyroid nodules are discovered as incidental findings on imaging studies performed for other reasons [5].

Workup of a thyroid nodule includes laboratory testing, ultrasonography, and biopsy, if indicated. The main concern is to exclude malignancy [6]. There have been great improvements in thyroid ultrasonography and ultrasound-guided thyroid fine-needle aspiration cytology (FNAC) [7], resulting in an increase in papillary thyroid microcarcinomas (PTMC), defined as PTC ≤ 1 cm [7]. In 1988–1989, only 25% of newly documented thyroid tumours were less than or equal to 1 cm, in comparison to 39% of the new thyroid cancer diagnoses in 2008–2009 [8]. The American Thyroid Association (ATA) recommends the use of FNA on thyroid nodules ≥ 1.5 cm in the presence of a low suspicion sonographic pattern and nodules ≥ 1 cm in the presence of intermediate or high suspicion patterns, effectively leading to active surveillance of smaller nodules [6, 9]. Currently, low risk biopsy-proven PTMC are thought to be indolent tumours that can be treated with surgical excision or active surveillance [6, 9]. A small subset of PTMC exhibits aggressive characteristics associated with true malignant behavior, including local and distant recurrences [10]. Identifying these tumours early on can improve the outcome. However, many of these aggressive features can only be identified post-operatively on histologic specimens.

Molecular testing is an additional tool that is being explored in thyroid cancer diagnostics to overcome this knowledge gap. Certain mutations have been linked to more aggressive tumour behaviour and molecular testing can assist in the interpretation of findings and the selection of an appropriate treatment plan [6]. The *BRAF V600E* mutation is the most commonly reported mutation in patients with PTC and PTMC [11]. The prevalence of *BRAF V600E* mutation varies significantly according to the histotype of PTC (30–90% of cases), being the highest in the tall cell variant of PTC (up to 95%) which is the most common aggressive variant of PTC. Activation of the wild type (*wt*) *BRAF* kinase is tightly regulated in the mitogen activating protein kinase (MAPK) pathway. The valine to glutamate mutation in the 600th position (V600E) of the peptide sequence results in constitutive activation of the *BRAF* kinase, resulting in oncogenesis [12]. While there is some controversy on this topic, many studies have reported that PTC with mutated *BRAF V600E* is associated with a poorer prognosis [13–23]. Since *BRAF V600E* has been linked to aggressive behaviour in PTC, the goal of this study is to determine if the presence of *BRAF V600E* in PTMC and small PTC is predictive of aggressive disease that requires more aggressive treatment than active surveillance or thyroid lobectomy.

## Materials and methods

### Study design

This is a retrospective chart review of 121 patient charts performed at a McGill University teaching hospital in Montreal, Quebec, Canada. The protocol was approved by the Medical-Bioethics Research Ethics Committee (REC) of the Integrated Health and Social Services Network for West-Central Montreal. Data on baseline patient demographics and characteristics, preoperative FNAC results, molecular mutation testing, and postoperative pathology was collected.

### Patient selection

Patients included in this study underwent a thyroid FNAC and subsequent surgery at our institution between 2017 and 2020. Charts were reviewed if the patient underwent thyroid surgery for PTMC (≤ 1 cm) or small PTC (1–1.5 cm) and molecular testing for *BRAF V600E* mutation was completed on these nodules. A limited ipsilateral central neck dissection was performed for all patients unless gross lymphadenopathy was visualized on pre-operative imaging or intraoperatively. In this case, a more formal central neck dissection was performed. The McGill Thyroid Nodule Score (MTNS) was calculated for all patients. This scoring system was used as it is very diverse, incorporating patient demographics, physical examination, imaging, and cytologic features to predict risk of thyroid cancer in the nodule. Patients with benign tumours on final pathology or who had undergone molecular testing that did not test for *BRAF V600E* mutation were excluded.

Two study groups were created based on tumour size: PTMC and small PTC. The groups were analysed for the presence of *BRAF V600E* mutation and aggressive features. Experienced thyroid pathologists reviewed all samples and reported aggressive features. Aggressive features noted on postoperative pathology reports included: macroscopic extrathyroidal extension (ETE), lymph node metastasis (LNM), and high-risk histological features (tall cell, columnar cell, hobnail/micropapillary, solid/trabecular, and diffuse sclerosing).

### Statistical analysis

Descriptive statistics were performed. Fisher exact tests, *t* tests and Mann–Whitney *U* tests were performed to calculate statistical significance between groups, using a threshold *p* < 0.05 to identify significant differences. These statistical analyses were performed using Microsoft Excel. Relative risk calculations were performed using MedCalc software.

## Results

A total of 121 patient charts fit the selection criteria and were included in this study. The 121 patients were subdivided into two study groups based on tumour size: PTMC (n = 55) and small PTC (n = 66). No statistically significant difference was detected between PTMC and small PTC with regards to age (*p* = 0.32), gender (*p* = 0.66), Bethesda diagnostic category (*p* = 0.87), or MTNS (*p* = 0.31).

All patients underwent preoperative molecular testing for *BRAF V600E*. In *BRAF V600E* mutated patients, there was a significantly higher MTNS (*p* < 0.001) and a significantly higher Bethesda score distribution (*p* < 0.001). These findings were consistent within the PTMC (*p* = 0.0042) and small PTC (*p* < 0.001) size groups. The baseline characteristics are reported in Table 1.

**Table 1:** Baseline characteristics

Tumour size	*BRAF*	Mean age	Gender (F:M)	Mean MTNS	Bethesda diagnostic category (score) distribution (%)			
					III	IV	V	VI
PTMC (≤ 1 cm)	*V600E*	47.7	3.8	13.5*	4.2	0.0	37.5	58.3
	*wt*	45.8	4.2	10.5	6.5	32.3	35.5	25.8
Small PTC (1.1–1.5 cm)	*V600E*	46.7	2.5	14.7*	3.6	0.0	28.6	67.9
	*wt*	49.1	3.2	10.4	18.4	26.3	31.6	23.7
All tumours (≤ 1.5 cm)	*V600E*	47.4	3.0	14.1*	3.8	0.0	32.7	63.5
	*wt*	47.7	3.6	10.5	13.0	29.0	33.3	24.6

MNTS, McGill Thyroid Nodule Score; PTMC, papillary thyroid microcarcinoma; PTC, papillary thyroid carcinoma; *wt*, wild type

^*^Statistically significant *p* value < 0.05

**Fig. 1:**
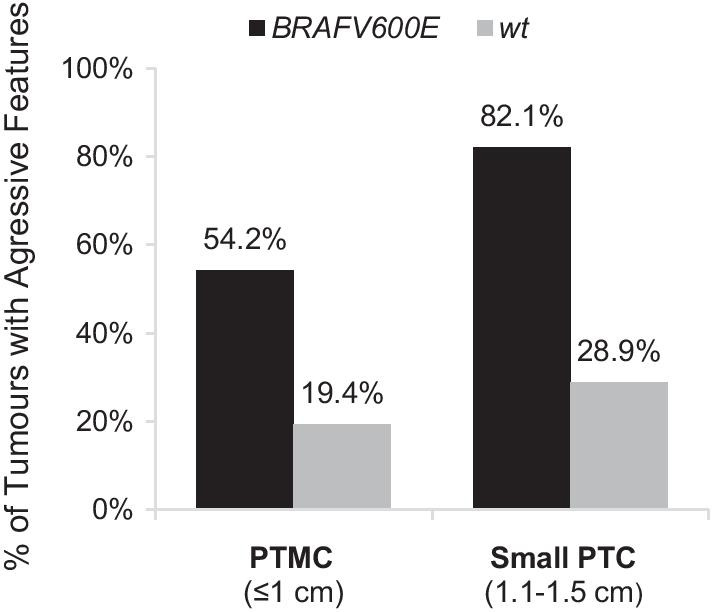
Percentage of papillary thyroid microcarcinoma (PTMC, ≤ 1 cm) and small papillary thyroid carcinoma (PTC, 1.1–1.5 cm) demonstrating aggressive tumour features, subdivided by *BRAF V600E* mutation status

Subdivided by size of tumour, the *BRAF V600E* mutation was detected in 43.6% of PTMCs and 42.4% of small PTCs. Of the mutated PTMC nodules, 54.2% demonstrated aggressive characteristics on final pathology as compared to 19.4% of the wild type PTMCs (*p* = 0.010). Of the mutated small PTC tumours, 82.1% had aggressive features. In contrast, 28.9% of the wild type small PTCs showed aggressive features (*p* < 0.001) (Fig. 1).

Aggressive features noted on final pathology are further characterized in Table 2. In the PTMC group, significant differences were detected with regards to having a tall cell variant (*p* = 0.031) and more than one aggressive feature (*p* = 0.012), with a maximum of two features in five tumours. In the small PTC group, significant differences were found among the occurrence of LNM (*p* = 0.0016), tall cell variants (*p* = 0.0011), and having more than one aggressive feature (*p* = 0.0034) on final pathology. Of the mutated small PTC tumours, six had two aggressive features and two had three aggressive features. One wild type PTC tumour had two aggressive features.

**Table 2:** Aggressive tumour features

	PTMC (≤ 1 cm)		Small PTC (1.1–1.5 cm)	
BRAF	*V600E* (n = 24)	*wt* (n = 31)	*V600E* (n = 28)	*wt* (n = 38)
ETE	4 (16.7%)	1 (3.2%)	4 (14.3%)	1 (2.6%)
LNM	10 (41.7%)	5 (16.1%)	16 (57.1%)*	7 (18.4%)
**High-risk variants**				
Tall cell	4 (17.4%)*	0	11 (39.3%)*	2 (5.3%)
Columnar	0	0	0	0
Hobnail/Micropapillary	0	0	2 (7.1%)	0
Solid/Trabecular	0	0	0	1 (2.6%)
Diffuse sclerosing	0	0	0	1 (2.6%)
> 1 aggressive feature	5 (20.8%)*	0	8 (28.6%)*	1 (2.6%)
Total Aggressive	13 (54.2%)*	6 (19.4%)	23 (82.1%)*	11 (28.9%)

PTMC, papillary thyroid microcarcinoma; PTC, papillary thyroid carcinoma; *wt*, wild type; ETE, extrathyroidal extension; LNM, lymph node metastasis

^*^Statistically significant *p* value < 0.05

Risk ratios of displaying aggressive tumour features were calculated (Table 3). Overall, the risk ratio of having aggressive disease with a detected *BRAF V600E* mutation was 2.81 (CI 1.79–4.41, *p* < 0.001). When subdivided by size, the risk ratio was 2.84 (CI 1.25–6.27, *p* = 0.012) for PTMC and 2.84 (CI 1.67–4.81, *p* < 0.001) for small PTC.

**Table 3:** Relative risk of aggressive tumour features in *BRAF V600E* mutated papillary thyroid tumours

	Relative risk	95% CI	*p* value
PTMC (≤ 1 cm)	2.80	1.25–6.27	0.012*
Small PTC (1.1–1.5 cm)	2.84	1.67–4.81	< 0.001*
All (≤ 1.5 cm)	2.81	1.79–4.41	< 0.001*

PTMC, papillary thyroid microcarcinoma; PTC, papillary thyroid carcinoma; CI, confidence interval

^*^Statistically significant *p* value < 0.05

## Discussion

The role of *BRAF* kinase in oncogenesis has been widely established [12]. Constitutive activation of *BRAF* through mutagenesis upregulates the mitogen activating protein kinase (MAPK) signalling pathway resulting in cell proliferation [24]. *BRAF V600E* is the most common mutation in PTC and in PTMC [25–27]. Previous studies have described an association of *BRAF V600E* mutation with aggressive features of PTC, including macroscopic extrathyroidal extension (ETE), lymph node metastasis (LNM), advanced stage, and disease recurrence [13–17, 21–23, 28]. In addition, the prevalence of *BRAF V600E* mutation is the highest in some aggressive histologic variants of PTC such as the tall cell variant (up to 95%) and the hobnail/micropapillary variant (70–80%). Currently, there is debate surrounding the implications of *BRAF V600E* on prognostic factors of PTMC, and whether *BRAF V600E* status should be used to guide management [9, 22, 29]. When taken in isolation, *BRAF V600E* has a low positive predictive value for detecting PTMC that will progress and spread outside of the thyroid. There is still limited data to indicate that any molecular findings, such as *BRAF V600E* mutation, should impact the suitability of a small PTC for active surveillance. However, some authors suggest that long-term active surveillance may not be a good alternative to surgery for patients with *BRAF V600E*-positive low-risk PTMC [22, 29]. The extensive Japanese, Korean, and more recent North American experiences have not used molecular markers as inclusion or exclusion criteria for active surveillance.

Guidelines on management of thyroid nodules have been set out by multiple organizations. The ATA recommends FNAC based on sonographic findings. A FNAC should be performed on nodules ≥ 1 cm in the presence of intermediate or high suspicion patterns and nodules ≥ 1.5 cm in the presence of a low suspicion sonographic pattern [6]. In general, if a FNAC is diagnostic for papillary thyroid malignancy, surgery is the recommended treatment. Recommendation #12 of the 2015 ATA guidelines proposes active surveillance as an alternative to immediate surgical excision in patients with a low-risk tumor (specifically citing PTMC with no high risk features as an example), comorbid patients with high surgical risk, patients with short remaining lifespans, or patients with other medical or surgical issues to address prior to thyroid surgery. This addition to the ATA guidelines is based on findings from two prospective studies in Japan that demonstrated disease specific mortality, loco-regional recurrence and distant recurrence rates similar to patients who underwent immediate surgery. As well, patients who eventually needed surgical excision did not suffer further negative consequences, suggesting that the delayed surgery did not affect the outcome [9, 30, 31]. Despite this evidence from Japan, active surveillance is not often implemented in North America. This may be because physicians and patients alike are uncomfortable as there are patients within this low-risk PTMC group that develop loco-regional or distant metastases [32]. There are no clinical or radiological features that can consistently identify the minority of PTMC patients who will have significant progression.

If surgery is selected to treat PTMC, the ATA recommends lobectomy for low-risk nodules (no ETE, LNM, prior irradiation, or family history of thyroid cancer)[6]. Suspicious lymphadenopathy can be at times detected preoperatively using ultrasound. However, in approximately half of cases, LNM is noted intra-operatively or post-operatively on pathology and sometimes results in the need for completion surgery to remove the rest of the thyroid gland [33, 34].

Prognostic factors of PTMC have been previously explored, patients at high risk of recurrence are those with positive lymph node metastases [10, 28, 35–37], patients with hoarseness which signifies recurrent laryngeal nerve involvement [37], and tumor multifocality [10, 28]. Aggressive variants of PTC such as tall cell and hobnail may also be associated with recurrence and less favourable outcomes, but these features are difficult to identify preoperatively on FNAC and are usually reported after examination of the surgical pathology specimen. This study postulates that molecular testing for *BRAF V600E* can prove to be an important approach to identifying high-risk small thyroid nodules and therefore guide initial surgical management.

This study analyzed data from two groups of small thyroid nodules: PTMC (≤ 1 cm) and small PTC (1–1.5 cm). The two size categories were selected to follow up previous research in the field. Specifically, the study carried out by Tuttle et al. defined low risk PTC as nodules ≤ 1.5 cm in size and compared the pathologic characteristics of these two groups (≤ 1 cm) and (1–1.5) [38]. They found that tumor size at the time of diagnosis did not correlate to overall growth in diameter or total volume. In another study by Sakai et al., the inclusion criteria for active surveillance were expanded to T1bN0M0, meaning including nodules ≤ 2 cm with no nodal or distant metastases [39].

In this study, no significant difference was observed between these size groups in patient demographics (age, gender), MTNS, Bethesda score distribution, or incidence of *BRAF V600E* mutation. The *BRAF V600E* mutation was associated with aggressive tumour features in both PTMC (*p* = 0.011) and small PTC (*p* < 0.001). In the PTMC group, *BRAF V600E* tumours were found to have a significantly greater occurrence of tall cell variants (*p* = 0.031) and having greater than one aggressive feature (*p* = 0.012). In the small PTC group, *BRAF V600E* tumours had a significantly greater occurrence of lymph node metastases (*p* = 0.0016), tall cell variants (*p* = 0.0011), and greater than one aggressive feature (*p* = 0.0034).

Our findings demonstrate that *BRAF V600E* mutation is associated with aggressive disease in PTC ≤ 1.5 cm (risk ratio 2.81, CI 1.79–4.41). *BRAF V600E* mutated PTMC (risk ratio 2.80, CI 1.25–6.27) and small PTC (risk ratio 2.84, CI 1.67–4.81) were more likely to display aggressive features such as ETE, LNM, and high-risk histological variants.

This study had several limitations. As a single-center study taking place in urban Montreal, this study is subject to geographic selection bias. Future studies should include patients from multiple institutions. In addition, there is another selection bias since molecular testing for *BRAF V600E* performed for this study was paid for by the patient. Therefore, not all patients with PTC ≤ 1.5 cm had molecular testing performed. Also, the large majority of thyroid nodules ≤ 1.5 cm were not biopsied. As a result, more aggressive nodules which may be more likely to harbour a *BRAF V600E* mutation may be overrepresented in this series. This study only tested for the *BRAF V600E* mutation and did not control for other mutations. There is evidence suggesting other driver mutations in combination with other mutations including *BRAF V600E*, such as *TP53* [40] and *TERT* mutations (41, 42), contribute even more to an aggressive tumour phenotype. Future studies should include these mutations into the analysis. Lastly, this study is limited by its retrospective nature. Disease recurrence data was not included in this study as there was no patient follow-up, however, future studies looking at this study’s population may be studied in the future to determine recurrence rates, additional treatment required versus resolution.

## Conclusion

In this study, *BRAF V600E* mutation was associated with aggressive disease in small papillary thyroid carcinoma ≤ 1.5 cm and papillary thyroid microcarcinoma ≤ 1 cm, defined by: ETE, LNM, and high-risk histological variants (tall cell, columnar, solid/trabecular, diffuse sclerosing). The findings demonstrate the importance of molecular testing for the *BRAF V600E* mutation in PTMC and small PTC. Molecular testing can help guide therapeutic decision-making and identify high risk PTMC and small PTC that require surgical intervention over clinical monitoring.

## Supplementary Information

**Additional file 1**. Appendix 1: Full data set of 121 patients included in the study.

## Data Availability

All data generated or analyzed during this study are included within the article (and its additional files).

## Abbreviations

ATA: American Thyroid Association
ETE: Extra-thyroidal extension
FNAC: Fine needle aspiration cytology
LNM: Lymph node metastasis
MTNS: McGill Thyroid Nodule Score
PTC: Papillary thyroid carcinoma
PTMC: Papillary thyroid microcarcinoma
*wt*: Wild-type
